# A PKS/NRPS/FAS Hybrid Gene Cluster from *Serratia plymuthica* RVH1 Encoding the Biosynthesis of Three Broad Spectrum, Zeamine-Related Antibiotics

**DOI:** 10.1371/journal.pone.0054143

**Published:** 2013-01-17

**Authors:** Joleen Masschelein, Wesley Mattheus, Ling-Jie Gao, Pieter Moons, Rob Van Houdt, Birgit Uytterhoeven, Chris Lamberigts, Eveline Lescrinier, Jef Rozenski, Piet Herdewijn, Abram Aertsen, Chris Michiels, Rob Lavigne

**Affiliations:** 1 Division of Gene Technology, KU Leuven, Heverlee, Belgium; 2 Laboratory of Food Microbiology, KU Leuven, Heverlee, Belgium; 3 Interface Valorisation Platform, KU Leuven, Leuven, Belgium; 4 Laboratory for Medicinal Chemistry, Rega Institute for Medicinal Research, Leuven, Belgium; University of Groningen, The Netherlands

## Abstract

*Serratia plymuthica* strain RVH1, initially isolated from an industrial food processing environment, displays potent antimicrobial activity towards a broad spectrum of Gram-positive and Gram-negative bacterial pathogens. Isolation and subsequent structure determination of bioactive molecules led to the identification of two polyamino antibiotics with the same molecular structure as zeamine and zeamine II as well as a third, closely related analogue, designated zeamine I. The gene cluster encoding the biosynthesis of the zeamine antibiotics was cloned and sequenced and shown to encode FAS, PKS as well as NRPS related enzymes in addition to putative tailoring and export enzymes. Interestingly, several genes show strong homology to the pfa cluster of genes involved in the biosynthesis of long chain polyunsaturated fatty acids in marine bacteria. We postulate that a mixed FAS/PKS and a hybrid NRPS/PKS assembly line each synthesize parts of the backbone that are linked together post-assembly in the case of zeamine and zeamine I. This interaction reflects a unique interplay between secondary lipid and secondary metabolite biosynthesis. Most likely, the zeamine antibiotics are produced as prodrugs that undergo activation in which a nonribosomal peptide sequence is cleaved off.

## Introduction

Polyketides (PKs) and nonribosomal peptides (NRPs) represent two large families of structurally diverse and complex microbial metabolites that include many therapeutically valuable antibacterial drugs. Both are synthesized with similar logic by large multifunctional enzymes known as polyketide synthases (PKSs) and nonribosomal peptide synthetases (NRPSs), which link simple monomeric building blocks (acyl-CoAs and amino acids, respectively) by a cascade of condensation reactions. The enzymes are organized in a modular assembly line fashion, in which each module consists of a set of enzymatic domains, responsible for one discrete round of chain elongation and a variable set of modifications on each intermediate [1], [2]. The modular architecture and the functional versatility of these enzymes, combined with post-PKS tailoring reactions, generate stereochemically complex compounds, characterized by a high level of structural variation. Depending on their biochemical properties and molecular architecture, PKSs are subdivided into two main classes. Type I PKSs consist of one or more multifunctional enzymes with active sites for each separate enzymatic reaction in the pathway. In contrast, type II synthases work iteratively, being composed of a minimal set of individual proteins that work together to create aromatic compounds [3].

Because of their structural and functional similarities, PKSs and NRPSs can form mixed assembly lines which give rise to hybrid polyketide-peptide natural products, as exemplified by epothilone D, rapamycin, yersiniabactin, and kalimantacin [4]–[7]. In addition, PKSs have also been shown to form hybrid assembly lines with the evolutionary related fatty acid synthases (FASs) with which they share striking similarities in domain organization, catalytic reactions, precursor usage and overall architectural arrangement. Recently, large-scale analysis of sequenced microbial genomes has revealed a variety of iteratively acting FAS/PKS hybrid systems closely related to the PUFA (polyunsaturated fatty acid) synthases [8]. The *pfaEABCD* gene cluster is responsible for the production of long-chain polyunsaturated fatty acids such as eicosapentaenoic acid (EPA) and docosahexaenoic acid (DHA) in marine bacteria [9]. The related FAS/PKS gene clusters, however, do not produce EPA or DHA but manufacture various other long chain fatty acyl products typically consisting of more than 20 carbons. Next to the type I fatty acid synthase (FASI) and the type II FAS system (FASII), whose structural organization served as the basis for the classification of PKSs, the iterative FAS/PKS mechanism represents the third bacterial fatty acid biosynthesis mechanism, referred to as “secondary lipid biosynthesis” [8].

*Serratia plymuthica* RVH1 is an opportunistic Gram-negative bacterium with strong biofilm-forming capacity, isolated from an industrial food processing environment [10]. The strain is characterized by a LuxI/LuxR type quorum sensing (QS) system SplI/SplR that regulates the production of a number of extracellular enzymes [11], the switch from mixed acid fermentation to butanediol fermentation [12], [13], and the production of an antimicrobial compound [11] which is the research focus here. This compound is active against a broad spectrum of Gram-negative and Gram-positive bacteria and no cross-resistance to known antibiotics has been observed [14]. Moreover, it has been shown to structurally differ from other known antimicrobials produced by *Serratia* strains [15]. The production of the antibiotic has been shown to provide a selective advantage to *S. plymuthica* RVH1 in competition with other bacteria, e.g. in mixed biofilms [16].

**Figure 1:**
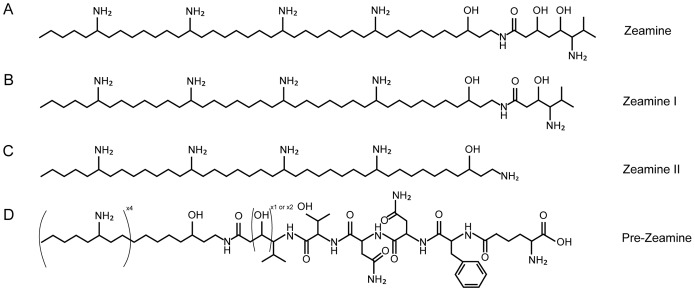
Overview of the zeamine antibiotics. Chemical structures of A) zeamine, B) zeamine I and C) zeamine II isolated from *S. plymuthica* strain RVH1. D) Proposed structure for the pre-zeamine antibiotics.

Here, we provide evidence that the antimicrobial activity of *S. plymuthica* RVH1 stems from the production of the broad spectrum polyamino antibiotics identified as zeamine and zeamine II [17], [18] (Figure 1A, 1C) and a previously uncharacterized analogue designated zeamine I (Figure 1B). Also, we report the cloning, sequencing and analysis of the gene cluster responsible for zeamine biosynthesis and propose that an unprecedented interaction between a hybrid PKS/NRPS and an iterative FAS/PKS system is responsible for the biosynthesis of the zeamine antibiotics.

## Results

### Isolation and Structure Determination of the Antibiotic Compounds from *S. plymuthica* RVH1

During a screening of 68 Gram-negative strains, isolated from an industrial food processing line, broad spectrum antimicrobial activity was detected in one of the strongest biofilm-forming isolates designated *Serratia plymuthica* RVH1 [15]. The optimal growth temperature of the strain is 30°C whereas fermentation experiments showed that antibiotic production is highest at 16°C (data not shown). Since the production of the antimicrobial compound in RVH1 is quorum sensing-regulated [11], fermentation cultures were supplemented with synthetic N-(3-oxo-hexanoyl)-C_6_-homoserine lactones which made it possible to achieve a yield of 20 mg/l of over 90% pure compounds. Using the *S. aureus* lawn plate assay, crude extracts were purified by silica gel chromatography leading to the isolation of the antimicrobial compounds as a yellow solid. LC/MS analysis of the purified compounds revealed two overlapping peaks (retention times: 4.05 and 4.10) with m/z of 654, and 797 and 841 respectively (Figure S1). Since the compound with m/z = 654 eluted faster than the compounds with m/z = 797 and 841, it was possible to separate the latter two compounds from the first. High-resolution mass spectrometry analysis (HRMS) (Figure S2) of these two compounds showed molecular peaks at [M+H]/e = 841.81913 and [M+H]/e = 797.79272 corresponding to molecular formulae of C_49_H_104_N_6_O_4_ and C_47_H_100_N_6_O_3_ respectively (<1 ppm error). The first molecular formula is identical to that of zeamine, isolated from *Dickeya zeae* DZ1 (Figure 1A) [17], [18]. ^1^H-NMR and ^13^C-NMR data, combined with ^1^H,^13^C – HSQC, ^1^H,^13^C – HSQC-TOCSY, ^1^H,^13^C – HMBC and DQFCOSY analysis (Figures S3, S4, S5, S6, S7) confirmed that the structure is identical to zeamine which means that the compound with m/z = 654 must be zeamine II ([M+H]/e = 654, 6993) (Figure 1C) [17], [18]. The compound with a molecular peak at [M+H]/e = 797.79272 represents an as yet uncharacterized compound which resembles zeamine and which we therefore designate as zeamine I (Figure 1B). MS-MS (Figure S8) combined with detailed NMR analysis revealed that the conserved polyamino part of zeamine I is connected via an amide bond to a 4-amino-3-hydroxy-5-methylhexanoic acid moiety instead of 6-amino-3,5-dihydroxy-7-methyloctanoic acid in the case of zeamine. This is reflected in the disappearance of a signal in the ^13^C-NMR spectrum at 66 ppm and one at 40 ppm. The ^1^H-NMR spectrum is characterized by the loss of a multiplet at 4 ppm and one at 1.6 ppm. So far, overlapping retention times have hampered the separation of zeamine from zeamine I.

### Cloning, Identification and Sequencing of the Zeamine Biosynthetic Gene Cluster

To localize genes involved in zeamine biosynthesis, random Tn*5* transposon mutagenesis was carried out on *S. plymuthica* RVH1. Tn*5* insertion mutants with altered antibiotic production were identified by the soft agar halo method using *S. aureus* ATCC27661 as indicator strain. Of the 361 plACKO (promotorless lacZ Antimicrobial Compound Knock Out) mutants selected this way, 67 strains lost all antimicrobial activity, 127 strains showed reduced activity, and 5 strains exhibited increased antibiotic production. For nine mutants, the sites of Tn*5* insertion were identified by subcloning and sequencing. In addition to genes encoding efflux systems and a putative histidine kinase, transposon insertions were found in various polyketide synthase related genes and a nonribosomal peptide synthetase gene, suggesting a hybrid PKS-NRPS assembly line (Table S1). Since PKS and NRPS genes are usually organized in gene clusters, a genomic BAC library (3840 clones, 92-fold coverage) was constructed and subsequently PCR-screened using predicted polyketide and nonribosomal peptide–related genes identified from the transposon insertion sites as template for primer design (Table S2). Based on the results, combined with restriction analysis and BAC end sequencing, the BAC clone showing the most promise of containing the full gene cluster was subcloned and sequenced by means of shotgun sequencing and primer walking.

### Analysis of the Biosynthetic Gene Cluster

**Figure 2:**
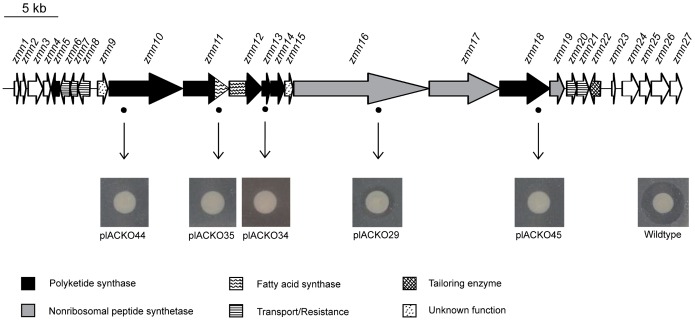
Genetic organization of the zeamine biosynthetic gene cluster and proposed role of individual genes. Positions of transposon insertions are marked by a dot. All but one, plACKO29, display a total loss of antibacterial activity compared to the wild type. All genes are transcribed in the same direction except for zmn5–8 and zmn22. Overlapping stop and start codons and the apparent lack of a transcriptional terminator in the short intergenic regions hints at translation coupling of these genes and suggests an operon-type organization.

This combined sequencing approach resulted in a total of 170 kb of contiguous BAC-clone sequence. Several of the identified Tn*5* transposon insertion sites were found to map to a 50 kb region encoding fatty acid, polyketide and nonribosomal peptide (FAS/PKS/NRPS) synthesis genes (Figure 2). With the exception of plACKO29, these transposon mutants all display a complete loss of antimicrobial activity compared to the wild type strain. The GC-content of the predicted polyketide/nonribosomal peptide operon ranges from 55% to 70% with an average of 61% which is comparable yet slightly higher than in the genus *Serratia* (52% to 60%) [19]. Blast database searches revealed the presence of an almost identical, unannotated region (99% identities, 99% similarities) in *Serratia plymuthica* AS12 (GenBank accession number: CP002774 ) [20], *Serratia plymuthica* AS9 (CP002773) [21] and *Serratia* sp. AS13 (CP002775).

Functional assignment predictions of each ORF in the FAS/PKS/NRPS region were made by comparing them with homologous sequences using BLAST (Table 1). Sequence analysis revealed 21 ORFs which include 5 PKS genes (*zmn10*, *zmn12*, *zmn13*, *zmn14* and *zmn18*), 3 NRPS genes (*zmn16*, *zmn17* and *zmn19*), 1 FAS/PKS gene (*zmn11*), 5 export related genes (*zmn6*, *zmn7*, *zmn8*, *zmn20* and *zmn21*) and genes with possible post-assembly tailoring functions (Figure 2) (EMBL nucleotide sequence database accession number: HE995400). Based on these predictions, it is proposed that *zmn5* and *zmn22* represent both ends of the predicted cluster.

**Table 1:** Proposed functions of open reading frames in the zeamine biosynthetic gene cluster region.

Gene	Size (aa)	Proposed function	Protein Homolog	Identity/Similarity(%/%)	Accession Number
zmn1	141	Hypothetical protein	CLDAP_13930	33/49	YP_005441330
zmn2	170	Hypothetical protein	Marme_2119	40/58	YP_004313199
zmn3	457	Pyridoxal-dependent decarboxylase	ACP_2295	61/74	YP_002755339
zmn4	217	Hypothetical protein	Tery_3479	25/45	YP_723042
zmn5	242	4′-phosphopantetheinyl transferase	SeW_A5143	63/72	ZP_02834856
zmn6	306	HlyD family secretion protein	ABI_18440	41/62	ZP_08263800
zmn7	239	ABC transporter ATP-binding protein	XsacN4_010100012101	56/67	ZP_09855363
zmn8	388	ABC transporter membrane protein	Smlt1652	42/61	YP_001971484
zmn9	331	Hypothetical protein	PAU_00912	49/69	YP_003039749
zmn10	2259	PKS	YfaA	67/77	YP_003039750
zmn11	1439	FAS/PKS	PfaC	69/81	YP_003039752
zmn12	1010	pfaD family protein	PAU_00916	79/88	YP_003039753
zmn13	255	3-oxoacyl-ACP reductase	XBJ1_2950	74/85	YP_003468838
zmn14	412	Thioester reductase	Ava_2595	31/49	YP_323105
zmn15	259	Carbon-nitrogen hydrolase	YafV	65/81	YP_003468836
zmn16	4169	NRPS	Bthur0013_24020	32/51	ZP_04072087
zmn17	2180	NRPS	Lilab_30101	42/58	YP_004668981
zmn18	1531	PKS	FJSC11DRAFT_1702	42/60	ZP_08985496
zmn19	439	Condensation domain-containing protein	BacA1	29/46	YP_005370175
zmn20	314	ABC transporter ATP-binding protein	STAUR_6062	45/63	YP_003955648
zmn21	371	ABC transporter permease	LBL_0136	33/54	YP_796690
zmn22	345	Hydrolase family protein	RHOER0001_1450	49/65	ZP_04383605
zmn23	106	NifA subfamily transcriptional regulator	Spro_2440	46/54	YP_001478669

### Genes Encoding FAS, PKS and NRPS

**Figure 3:**
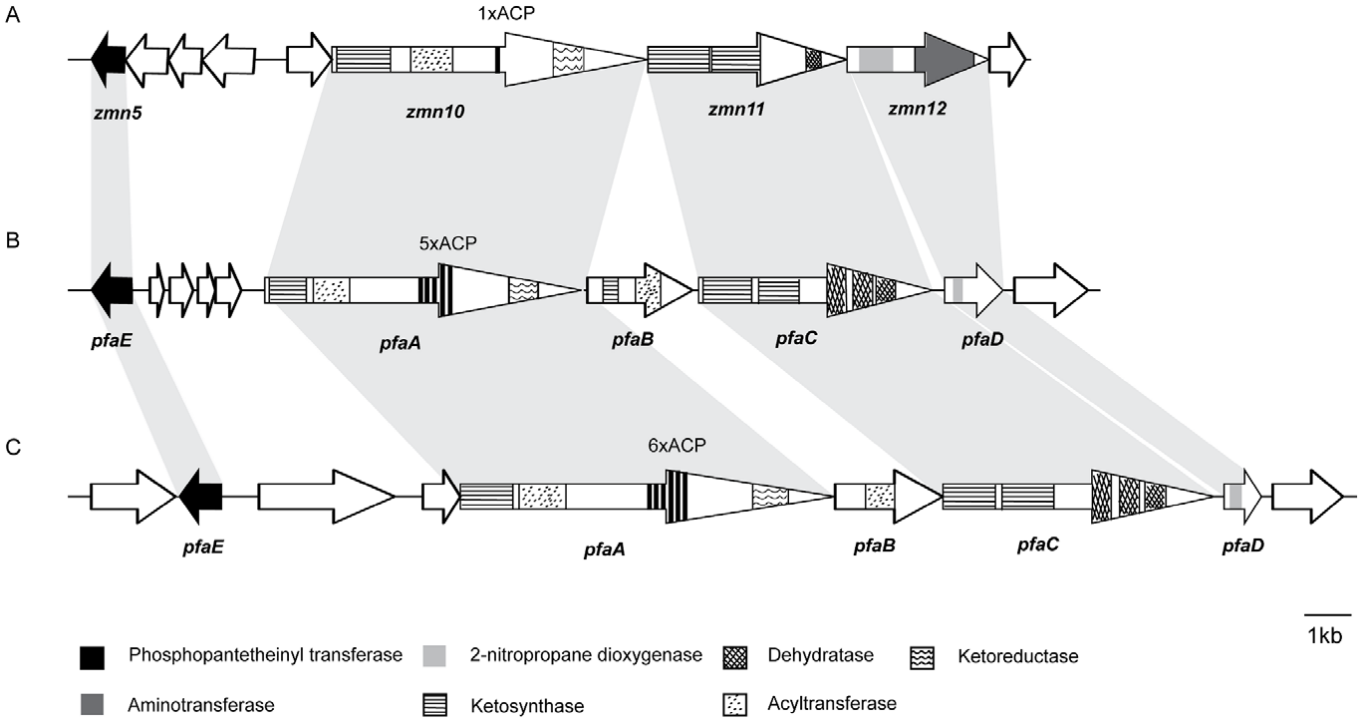
Pfa-like genes in the zeamine gene cluster. Comparison of A) part of the putative zeamine gene cluster from *S. plymuthica* RVH1 to the pfa clusters of genes responsible for biosynthesis of B) eicosapentaenoic acid (EPA) in *S. pneumatophori* SCRC-2738 and C) docosahexaenoic acid (DHA) in *M. marina* strain MP1(pDHA4) (C). Zeamine and pfa genes with corresponding functions and domain organization are connected by grey areas.

Three genes in the putative gene cluster (*zmn10*, *zmn11* and *zmn12*) show homology to the *pfa* clusters of genes involved in the biosynthesis of PUFAs in marine bacteria such as *Shewanella pneumatophori* SCRC-2738 [22], [23], *Moritella marina* MP-1 [24] and *Photobacterium profundum* SS9 [25]. In particular, the *pfaEABCD* operon encodes a FAS/PKS system responsible for the production of the long chain polyunsaturated fatty acids EPA and DHA and encodes multiple conserved enzymatic domains in an operon-type organization. *zmn10-12* share a common orientation, size, order, as well as domain structure (Figure 3) to *pfaA*, *pfaC* and *pfaD*. Zmn10 is similar to PfaA but unlike most PUFA synthases carries only one acyl carrier protein (ACP)-domain [9] Like PfaC, Zmn11 contains two KS domains and one FabA-like dehydratase/isomerase domain. Finally, Zmn12 contains an extra domain with putative aminotransferase activity in addition to the characteristic 2-nitropropane dioxygenase domain found in PfaD which acts as an enoyl reductase. This domain shows homology to a Class III pyridoxal phosphate (PLP)-dependent aminotransferase from *Streptococcus downei* F0415 (49% identities, 69% positives). No apparent *pfaB* ortholog is found in the vicinity of *zmn10-12*.

The involvement of a PfaA homologue (ZmsA) in zeamine biosynthesis has previously been reported by Zhou et al. [18]. Analysis of the partial zmsA sequence data available indicates significant sequence divergence between zmn10 and zmsA. Furthermore, both homologues contain a slightly different subset of domains.

A potential associated phosphopantetheinyl transferase (PPTase) (Zmn5) is encoded in close proximity upstream of the cluster. Analysis of conserved domains points out that this Sfp-type PPTase (surfactin phosphopantetheinyl transferase) is not a PUFA-specific PPTase (PfaE-like) but rather belongs to the other group of PKS/NRPS-specific PPTases (EntD-like) [26], [27]. However, all Sfp-type PPTases have been shown to enable PUFA biosynthesis [28]. Apart from the conserved P2 (GVDIE) and P3 (KESLFKA) motifs found in both groups of PPTases, Zmn5 is characterized by the PKS/NRPS-associated 1A (KRRADY), P1a’ (DRAP) and P1b’ (GSISH) domains instead of the PUFA-related P0, P1a and P1b domains (Figure S9) [26], [8].

Immediately downstream of the *pfaA*, *pfaC* and *pfaD* orthologs, a putative ketoreductase and thioester reductase are encoded (Zmn13 and Zmn14 respectively). Thioester reductases are typically located at the C-terminus of PKS/NRPS assembly lines and perform reductive chain termination. Zmn14 contains the characteristic R1 domain for NAD(P)H binding as well as the five other core motifs (R2–R6) found in reductase domains (Figure S10) [29].

Apart from the FAS/PKS genes related to PUFA synthases, sequence analysis revealed three other multifunctional genes that encode a type I PKS module consisting of a KS, AT, KR and ACP-domain (*zmn18*) and two modular NRPSs composed of the characteristic condensation – adenylation – thiolation (C-A-T) domain organization in addition to a putative epimerization domain in module 3 *(zmn16* and *zmn17)*. The initiation and the first three extension modules are located on Zmn16 whereas *zmn17* encodes a dimodular NRPS. Finally, *zmn19* is believed to encode of a free-standing condensation domain.

All predicted KS-domains in the cluster contain the C-H-H catalytic triad [30] except for the second KS domain of Zmn11 which is believed to be a chain length factor [25]. This hypothesis is supported here by the replacement of the conserved Cys residue in the active site by a Glu residue. Furthermore, the first active site His residue is replaced by Tyr (Figure S11A).

The two acyltransferase (AT) domains identified in Zmn10 and Zmn18 both contain the characteristic active site motif GXSXG (Figure S11B). Based on sequence comparison with database ATs with known substrate specificity, they are predicted to select malonyl-CoA [31], [32]. This is consistent with previous feeding experiments which revealed that zeamine, except for the amino isobutyl moiety, is completely derived from acetate units [17].

The three putative KR-domains identified in the cluster, contain the typical Rossman fold for NADPH cofactor binding as well as the conserved SYN residues (SYK for KR_Zmn18_) in the active site [33]. KR_Zmn10_ is characterized by the LDD-motif (but no apparent PxxxN motif) which correlates with B-type (“R configuration”) alcohol stereochemistry. The absence of this motif in KR_Zmn13_ and KR_Zmn18_ and the presence of the conserved tryptophan residue in KR_Zmn18_ is indicative for A-type KR-domains providing a hydroxyl group of the “S” configuration (Figure S11C) [33], [34].

The first two condensation domains of Zmn16 contain a HHxxxDA and HHxxxDE core motif respectively with significant similarity to the described conserved motif HHxxxDG [35] present in the third C-domain of Zmn16 and the first Zmn17 C-domain. In the final Zmn17 C-domain and in Zmn19, the first histidine is replaced by a cysteine residue (CHxxxDG) and a proline residue respectively (PHxxxDG). The sequence of the epimerization domain aligns well with the sequence of the condensation domains and contains the conserved core motifs (Figure S11D).

Building block specificity of the A-domains was predicted by the support vector machine (SVM)-based NRPSpredictor toolbox and are shown in Table 2 together with the deduced specificity-conferring codes [36], [37].

**Table 2:** Specificity-conferring codes of the adenylation domains from Zmn16 and Zmn17 and their predicted building block specificity.

Adenylation domain	Specificity-conferring code	Amino Acid Prediction
A1	DPRHLALLAK	2-Aminoadipic acid
A2	DTWTIASVSK	Phenylalanine
A3	DATKVGEVGK	Asparagine
A4	DATKVGEVGK	Asparagine
A5	DFWNIGMVHK	Threonine
A6	DALFIGGTFK	Valine

All T-domains contain the highly conserved 4′-phosphopantetheinyl binding site motif LGGXS [38], indicating their functionality (Figure S11E). This binding motif is conserved between PCP and ACP domains which undergo the same post-translational 4′-phosphopantetheinylation of the essential serine residue. ACP_Zmn10_ and ACP_Zmn18_ contain a slightly different motif (LGIXS and LNGXS respectively) (Figure S11F).

### Potential Tailoring, Resistance and Transport Genes

In addition to FAS, PKS and NRPS related genes, other putative gene functions possibly involved in tailoring, resistance or transport, were identified within the gene cluster.

The predicted gene product of *zmn15* belongs to the carbon-nitrogen hydrolase superfamily of enzymes (EC 3.5) that catalyze condensation and hydrolysis of a wide variety of non-peptide carbon-nitrogen bonds [39]. Members of this family include nitrilases, carbamylases, N-acyltransferases and amidases, all characterized by a α-β-β-α sandwich protein fold and classified in 13 branches according to their specific function and substrate specificity [40]. Based on the consensus motifs flanking the conserved catalytic triad Glu-Lys-Cys, Zmn15 can be classified as a putative branch 13 nitrilase. Suffering from a lack of functional information, this enzyme class is a collection of uncharacterized carbon-nitrogen hydrolases difficult to divide into specific similarity groups.

Five putative transport genes were identified within the cluster. The gene product of *zmn20* is listed as a polyamine-transporting ATPase in *Serratia* sp. AS12 (SerAS12_4273) whereas *zmn21* encodes a putative ABC-2 type transporter. This makes them likely candidates to export zeamine and its derivatives and/or confer self-resistance to *S. plymuthica* RVH1.

Like Zmn20 and Zmn21, Zmn7 and Zmn8 also form a putative ABC-transporter. *zmn7*, which encodes a putative ABC transporter associated ATP-binding protein, shows homology to a phosphonate-transporting ATPase from *Delftia* sp. Cs1-4 (DelCs14_0129, 52% identities, 66% similarities) and a macrolide-specific ABC-type efflux carrier from *Comamonas testosteroni* S44 (CTS44_21395, 54% identities, 67% similarities) whereas Zmn8 shares similarities with ABC-transporter permeases.

The deduced product of *zmn6* is a putative member of the HlyD family of secretion proteins. Together with HlyB, HlyD is responsible for the secretion of the hemolysin A toxic in uropathogenic *E. coli* via the type I secretion pathway [41].

Finally, a conserved domain search of the gene product of *zmn22* revealed the presence of an acyl-aminoacyl peptidase domain, belonging to the prolyl oligopeptidase family (EC 3.4.21.26). Acylaminoacyl peptidases, or acylpeptide hydrolases are known to catalyze the cleavage of short N-acylated peptides that play a role in a variety of important biological processes [42].

Four genes (*zmn1*, *2*, *4* and *9*) did not possess any significant similarity to sequences in the public databases and were indicated as hypothetical proteins.

## Discussion

Structural analysis in the current work revealed that *Serratia plymuthica* strain RVH1 produces two polyamino antibiotics with the same molecular structure as zeamine and zeamine II [17], along with a newly discovered analogue zeamine I (Figure 1C). Zeamine I shares the conserved polyamino tail with zeamine and zeamine II but differs in the valine-derived moiety. So far, the absolute stereochemical configuration of the zeamine antibiotics remains unknown.

The combination of random transposon mutagenesis and subsequent BAC-library screening led to the identification of the zeamine biosynthesis genes. Sequence analysis revealed that the gene cluster encodes fatty acid, polyketide as well as nonribosomal peptide biosynthetic enzymes together with predicted tailoring and export proteins. Interestingly, three genes (*zmn10*, *zmn11*, *zmn12*) are similar to genes in the *pfa* gene clusters (*pfaEABCD*) involved in the biosynthesis of long-chain polyunsaturated fatty acids in psychrophilic bacteria prevalent in deep-sea environments [22], [23], [24], [25], [43](Figure 3) Recent large-scale analysis of sequenced microbial genomes has revealed a wide variety of FAS/PKS gene clusters homologous to those responsible for LCPUFA synthesis in over ten microbial phyla. These related FAS/PKS gene clusters produce various specialized long chain fatty acids consisting of more than 20 carbons in a process referred to as “secondary lipid biosynthesis” [8]. The three zeamine genes share a common orientation, size, order, as well as domain organization with *pfaA*, *pfaC* and *pfaD* (Figure 3). A distinct feature of the zeamine FAS/PKS genes is the lack of a *pfaB* ortholog, nor is the corresponding AT-activity encoded in the *pfaC* ortholog (*zmn11*), as frequently observed in secondary lipid synthases [8]. Also, *zmn12* is the first *pfaD* ortholog reported to contain an aminotransferase domain. This domain belongs to the family of PLP-dependent aspartate aminotransferases (EC 2.6.1.1) and shows homology to glutamate-1-semialdehyde aminotransferases (EC 5.4.3.8). A similar PLP-dependent aminotransferase domain equally showing homology to glutamate-1-semialdehyde aminotransferases has previously been identified in MycA, a hybrid PKS/NRPS involved in the biosynthesis of the antibiotic mycosubtilin. Using glutamine as amine source, the MycA aminotransferase domain works *in cis* to incorporate an amine at the β-position of a growing acyl chain generating a β-aminothioester [44]. Immediately downstream of *zmn12*, a predicted ketoreductase and thioester reductase are encoded which show no apparent relationship to *pfa* genes.

Apart from FAS/PKS genes homologous to LCPUFA biosynthesis genes, an NRPS assembly line represents a noticeable part of the putative zeamine gene cluster. The NRPS genes encode a loading module and five extension modules which are predicted to activate and incorporate 2-aminoadipic acid, phenylalanine, asparagine (2×), threonine and valine in that order. A PKS gene encoding KS, AT, ACP and KR-domains (*zmn18*) and a stand-alone condensation domain (*zmn19*) are localized immediately downstream of the NRPS genes.

### Proposed Biosynthesis Model for the Zeamine Antibiotics

**Figure 4:**
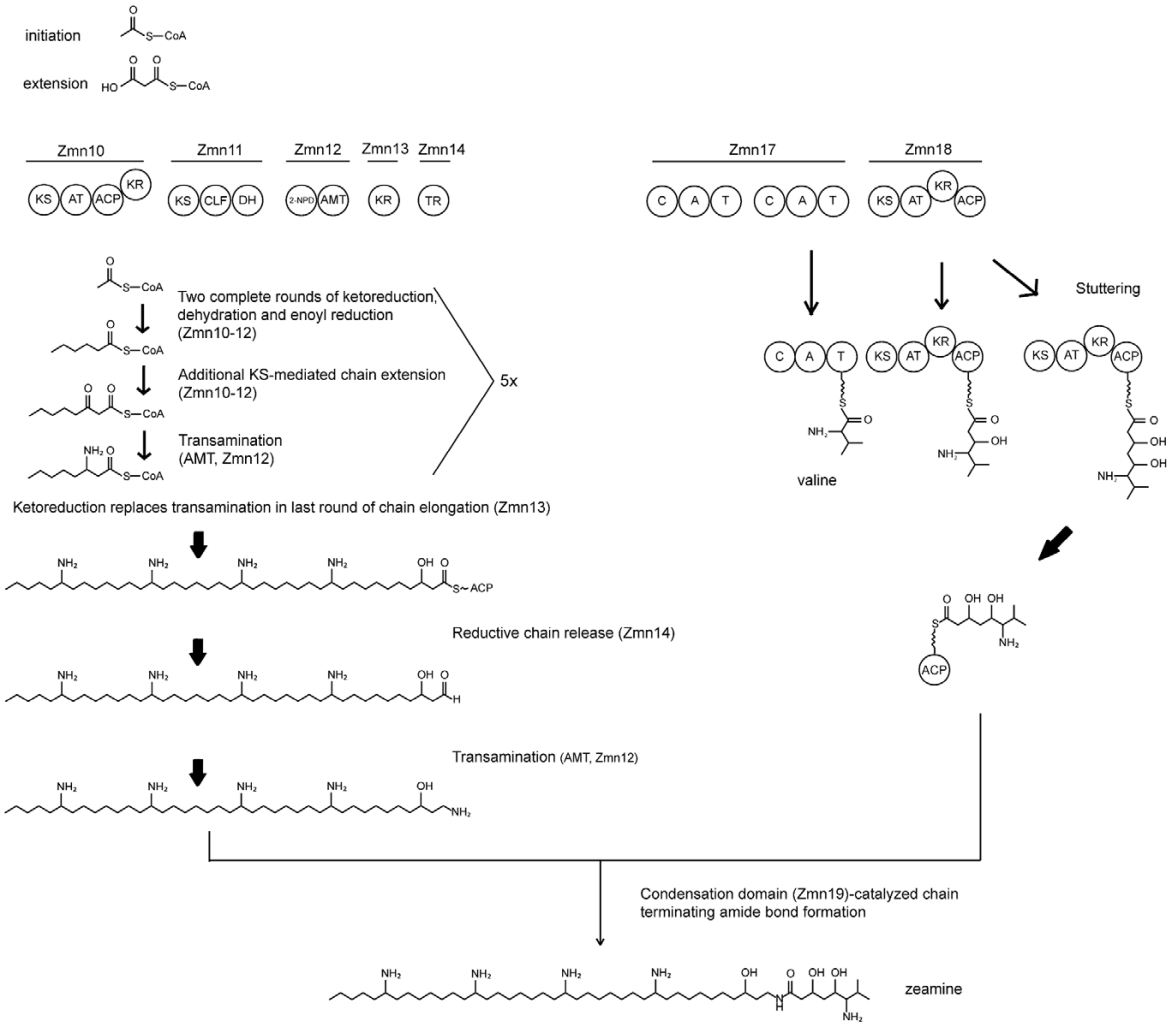
Proposed pathway for zeamine biosynthesis. Biosynthesis of the zeamine antibiotics can be envisaged as a two-part process. Zmn10-14 likely associate to form a multienzyme complex responsible for the coordinated formation of the polyamino tail in a process closely related to secondary lipid biosynthesis. On the other hand, the valine-derived moiety originates from the hybrid NRPS/PKS formed by Zmn16-18. In the case of zeamine and zeamine I, both parts are connected post-assembly by means of chain-terminating amide bond formation, catalyzed by Zmn19. In the course of zeamine biosynthesis, a nonribosomal pentapeptide sequence is likely cleaved off, resulting in the observed zeamine structures.

Bio-informatical and experimental analysis of the genes in the putative gene cluster allow us to overhaul, expand and specify the pathway for zeamine biosynthesis postulated by Wu et al. [17] (Figure 4). We hypothesize that a mixed FAS/PKS and a hybrid PKS/NRPS each synthesize parts of zeamine which are linked together post-assembly in the case of zeamine and zeamine I. Analogous to the PUFA synthases, the multidomain FAS/PKS-related enzymes encoded by the zeamine biosynthesis gene cluster very likely catalyse the repeated steps in the production of the polyamino chain, in which each condensation reaction is followed by a complete round of ketoreduction, dehydration/isomerization and enoyl reduction. In selected cycles however, the reductive reaction cycle is replaced with a single transamination step, possibly catalyzed by the aminotransferase domain present in Zmn12, similar to the role of the aminotransferase domain identified in MycA [45]. In the last round of elongation, the transamination step is replaced by a ketoreduction reaction. Zmn14-catalyzed reductive chain termination would result in an aldehyde which is the substrate for a final transamination reaction. In the biosynthesis of the siderophore myxochelin B, a similar chain termination procedure is observed, in which the aldehyde aminotransferase MxcL, catalyses the exchange of the amino group [46]. In the zeamine gene cluster, the aminotransferase domain present in Zmn12 is believed to fulfill this role.

The valine-derived moiety is proposed to originate from the hybrid PKS/NRPS assembly line formed by Zmn16-18. We predict that the amino acid valine, incorporated by the C-terminal NRPS module, acts as a primer for two additional rounds of PKS chain elongation with KR-mediated reduction of the β-ketogroup, catalyzed by Zmn18. This would generate 6-amino-3,5-dihydroxy-7-methyloctanoate-S-ACP which is subsequently coupled to the polyamino chain by formation of an amide bond. A likely candidate catalyzing this linkage is the free-standing condensation domain (Zmn19) encoded directly downstream of the PKS module. Condensation domain-catalyzed amide bond formation as alternative chain termination method has been reported in the biosynthesis of the siderophore vibriobactin and has been predicted to occur in several other NRPS including those of cyclosporine, enniatin, HC-toxin, PF1022A and vibriobactin. However, while chain termination of the latter examples involves intramolecular amide bond formation and subsequent cyclization, VibH has the ability to form an amide bond between a soluble substrate acting as the acceptor nucleophile and a carrier protein-bound acyl donor [47], [48].

Taking into account the antimicrobial activity of zeamine II, the connection of the polyamino chain to the 6-amino-3,5-dihydroxy-7-methyloctanoate does not seem to be crucial for its function as antibiotic [18]. The biosynthesis of zeamine could be envisaged as being the result of aberrant stuttering of Zmn19, a process previously observed in module 4 of 6-deoxyerythronolide B synthase and module 5 of the epothilone PKS [49], [50]. This would imply that iterative processing of the intermediate is kinetically more favorable than interprotein transfer to the free-standing downstream condensation domain. As such, Zeamine I can be regarded as being the ‘intended’ product of the biosynthesis cluster.

Remarkably, the products of the first five NRPS modules are not present in the final zeamine structure even though active site residues suggest assembly line functionality. Furthermore, the reduced antimicrobial activity of transposon mutant plACKO 29 (Figure 2) indicates a likely role of Zmn16 in the biosynthesis of zeamine although possible polar effects cannot be excluded in this respect. One could speculate that the zeamine antibiotics are produced as prodrugs (Figure 1D) that undergo post-assembly activation in which five amino acids are cleaved off. A similar process has been described for the xenocoumacin antibiotics. Here, the pre-xenocoumacins are trimmed periplasmatically upon secretion at an acylated D-Asn residue by XcnG, a membrane-bound peptidase, thereby turning inactive precursor molecules into the bioactive compound. Furthermore, there are indications that this process is conserved in several other biosynthetic gene clusters [51]. In the putative zeamine gene cluster, no XcnG homolog is found, however similar function may be exerted by Zmn22 predicted αβ hydrolase fold protein characterized by a predicted dipeptidyl aminopeptidase/acyl-aminoacyl peptidase domain. Ayg1p, a polyketide biosynthesis tailoring enzyme with a similar acyl-aminoacyl peptidase domain has been shown to hydrolytically cleave off acetoacetic acid from a heptaketide thereby giving rise to 1,3,6,8-tetrahydroxynaphthalene, the precursor of the fungal virulence factor melanin [52].

Alternatively, Zmn15, strategically encoded between the two biosynthesis machineries, belongs to the carbon-nitrogen hydrolase superfamily involved in amide-hydrolysis and condensation. Although some representatives of the family act on polypeptide side chains or carry out condensation reactions on the N-termini of proteins, so far no known members have been reported to cleave peptide bonds, making this enzyme an unlikely candidate for this cleavage reaction [38].

Apart from antibiotic properties, potent phytotoxic effects have also been attributed to the zeamine antibiotics in the phytopathogenic strain *Dickeya zeae* DZ1. More specifically, they have been shown to inhibit rice seed root and shoot germination [18]. Now, with *Serratia plymuthica* strain RVH1 and the rapeseed plant-associated *Serratia plymuthica* AS12 [20], AS13 and AS9 [21], four additional phylogenetically closely related, rhizosphere associated bacterial strains have been identified that harbor the zeamine biosynthesis genes. Therefore, it is plausible that horizontal gene transfer contributed to the spread of the zeamine-producing potential among plant-associated soil bacteria.

### Conclusion

In this study, we have identified the zeamine biosynthetic gene cluster from *Serratia plymuthica* RVH1, responsible for the production of three zeamine-related broad spectrum antibiotics. Analysis of the putative biosynthesis genes allowed us to propose a unique synergy between secondary lipid and secondary metabolite biosynthesis in which a mixed FAS/PKS, analogous to the PUFA synthases, and a hybrid NRPS/PKS each assemble parts of the zeamine natural products that are linked together post-assembly in the case of zeamine and zeamine I. Furthermore, indications are there that the compounds are initially synthesized as prodrugs that undergo an activation step in which a nonribosomal peptide sequence is cleaved off. The mechanism by which amino groups are incorporated into the zeamine backbone could offer prospects for the rational design of new bioactive compounds with increased functionality.

## Materials and Methods

### Bacterial Strains and Plasmids

*S. plymuthica* RVH1 served as the wild-type zeamine producer [15]. *Escherichia coli* Transformax EC100 and *E. coli* DH5α were used for BAC-library construction and routine subcloning purposes whereas *E. coli* S17-1 [53] was used for conjugative transfer of DNA to *S. plymuthica* RVH1. *Staphylococcus aureus* ATCC27661 was used in bioassays to monitor zeamine production. *E. coli sdiA::Km* [54], a kanamycin resistant strain unable to react on N-acyl-L-homoserine lactone signals, functioned as a test strain to identify random transposon mutants of *S. plymuthica* RVH1 with an altered production of zeamine. Bacterial strains and plasmids used during this work are listed in Table S3.

### Growth and Culture Conditions

*S. plymuthica* RVH1 was grown at 30°C in Lysogeny Broth (LB) for liquid cultivation and on LB agar (LB broth supplemented with 1.5% w/v agar) for solid cultivation. *E. coli* and *S. aureus* strains were grown at 37°C in LB broth and on LB-agar. When appropriate, media were supplemented with antibiotics at the following concentrations: kanamycin (50 µg/ml), ampicillin (100 µg/ml), chloramphenicol (50 µg/ml).

### Production and Isolation of Zeamine

*S. plymuthica* RVH1 was grown in liquid LB-medium, supplemented with 5 µM synthetic N-(3-oxo-hexanoyl)-C_6_-homoserine lactone (Sigma), at 16°C for 48 h on a rotary shaker (80 rpm). Cells were removed by centrifugation (13000 rpm, 20 min) after which the supernatant was adjusted to pH 10.0 with 1 M NaOH and extracted with n-butanol twice. After acidification to pH 3.0 with acetic acid, the extract was subjected to silica gel column chromatography (20×250 mm, MeOH/CH_2_Cl_2_ [1/8 to 1/4 to 1/2 to 1/1 to 1/0] followed by MeOH/H_2_O [8/1 to 4/1 to 4/1+200 mM NaCl]). Zeamine antibiotics eluted when NaCl was added to the solvent. LC/MS analysis was performed on a Finnigan Surveyor HPLC system using a C_18_ reverse phase column (Thermo Scientific HyPURITY 5 µm C_18_ 30×2.1 mm) and a solvent system consisting of MeOH in H_2_O supplemented with 0,1% formic acid (0–2 min 20%–95%, 2–3 min 95%, 3–4 min 95%-20%, 4–8 min 20%). Peaks were monitored with a mass detector (Finnigan LCQ Advantage Max ESI/MS).

### Structure Analysis of Zeamine

MS analysis was performed on a quadrupole/orthogonal acceleration time-of-flight mass spectrometer (Synapt G2 HDMS, Waters, Milford, MA). The instrument was operated at a resolution of 15000. Leucine enkephalin was used as reference for accurate mass measurements. The samples were dissolved in acetonitrile:water (1∶1) and infused at a flow rate of 5 uL/min. The capillary voltage was set to 3 kV. For NMR spectroscopy, samples were dissolved in 500 µl D_2_O with a final pH of 4.4. All spectra were recorded at 25°C on a Bruker Avance II 600 equipped with a 5 mm TCI HCN Z gradient cryoprobe. The Bruker Topspin 2.1 software was used to process the spectra. A 2D DQFCOSY [55] spectrum was measured with a spectral width of 6600 Hz in both dimensions using 64 scans and 512/2048 complex data points in t1 and t2, respectively. Heteronuclear correlation spectra were recorded using 64 scans and 1024/1024 complex data points and 200/11 ppm spectral widths in t1 and t2, respectively. The pulse sequence for the 2D gradient enhanced ^1^H-^13^C HSQC was as described by Sattler *et al.* [56]. The delays Δ1 and Δ2 in the inept transfer were set to 1.67 ms (1/4J_CH_). The 2D HSQC–TOCSY consisted of an HSQC building block [57] (Δ1 = Δ2 = 1.67 ms) followed by a clean MLEV17 TOCSY transfer step [58] during a mixing time of 60 ms prior to detection. Decoupling during the acquisition of the HSQC and HSQC–TOCSY spectra was achieved by using the garp sequence [59]. Sine-bell shaped gradients were applied along the z-axis during the sequences to obtain coherence selection and sensitivity enhancement. Prior to Fourier transformation, a squared sine-bell function was applied in both dimensions of 2D spectra.

### Bioassay for Zeamine Production

Zeamine production was monitored by bioassay. An LB agar plate was overlaid with a lawn of stationary phase *S. aureus* ATCC27661 cells, diluted to OD_600nm_ = 0,03 (5 µ/ml) and 10 µl samples of 18 h grown *S. plymuthica* RVH1 cultures were spotted on top. After incubation for 48 h at 16°C and 24 h at 30°C, the size of the inhibition zones was evaluated.

### *‘*Soft Agar Halo Method

After overnight incubation of indicator strains at 30°C in LB-medium, 50 µl of the stationary cultures was added to a petridish and mixed with LB soft agar (LB broth supplemented with 0.7% w/v agar). 5 µl of an overnight stationary culture of *S. plymuthica* RVH1 was spotted on top. To allow the growth of strain RVH1 before that of the test strains, the plates were incubated for 2 days at 16°C before further incubation at 30°C for 24 h. This way, a clear halo surrounded by a turbid lawn was visible around the RVH1 spot in case of a sensitive test strain.

### Random Transposon Mutagenesis and Identification of Genes Involved in Zeamine Biosynthesis

Random transposon mutagenesis was performed by transferring the suicide plasmid pUT mini-Tn5 *lacZ1* from *E. coli* S17-1 *λpir* to an *S. plymuthica* RVH1 *splI* insertion mutant which is deficient in quorum sensing [11], [60]. Using the soft agar halo method described above, mutant strains with reduced or absent growth inhibition zone were identified on LB soft agar using a kanamycin resistant test strain unable to react on signal molecules (*E. coli sdiA::Km)* and kanamycin as selection marker. Synthetic N-(3-oxo-hexanoyl)-C_6_-homoserine lactone (OHHL) signal was added to the plates to ensure maximal antibiotic production. To identify the site of transposon insertion, sonicated (Vibracell Sonics and Materials Inc.) genomic DNA of the selected mutants was ligated into *Sma*I digested pUC18 vectors and transformed into *E. coli* DH5α, followed by selection on LB-agar supplemented with ampicillin. The genes flanking the transposon insertions were identified by sequencing using the pUC24bis, pUC47 and KmKleckner primers (Table S2).

### Genomic Library Construction and Screening

BAC-library construction of *S. plymuthica* strain RVH1 was performed as described by Osoegawa *et al*. [61]. High-molecular-weight DNA was embedded in plugs, partially digested with *Hin*dIII and size selected by pulsed-field gel electrophoresis. Subsequent cloning in pIndigoBAC5 (Epicentre) vectors and transformation to *E. coli* Transformax™ EC100™ were performed as described by the manufacturer. This yielded a library of 3840 clones with an average insert size of 130 kb resulting in 92-fold coverage. Using the identified sequences flanking the transposon insertion sites as template for primer design, the library was screened by PCR to localize the zeamine biosynthesis genes. A *Hin*dIII restriction digest and BAC end sequencing using pINDIGO BAC-5 vector primers aided in selecting the best BAC clone for further analysis. For an overview of the oligonucleotide primers used in this study, see Table S2.

### DNA Sequencing and Analysis

The selected BAC-clone was subcloned using pUC19 vectors and sequenced by a combination of primer walking and shotgun sequencing. Sequencing reactions were run using Big Dye Terminator mix (Applied Biosystems), purified and analyzed on an ABI 3130 genetic analyzer. Sequence assembly into contigs was performed using Sequencher 4.8 software (Gene Codes Corporation, USA). ORF predictions were carried out by combining comparative genomics approaches (tBLASTx) and the ORF Finder (NCBI) and Genemark.hmm algorithms [62].

## Supporting Information

Figure S1 **LC-MS analysis of purified extract of** ***S. plymuthica*** **RVH1 containing zeamine, zeamine I and zeamine II.**
(PNG)

Figure S2 HRMS analysis of zeamine and zeamine I.
(PNG)

Figure S3 **^1^H-NMR spectrum of zeamine and zeamine I.**
(BMP)

Figure S4 **^13^C-NMR spectrum of zeamine and zeamine I.**
(BMP)

Figure S5 **^1^H,^13^C – HSQC spectrum of zeamine and zeamine I.**
(BMP)

Figure S6 **^1^H,^13^C – HSQC-TOCSY spectrum of zeamine and zeamine I overlayed with ^1^H,^13^C – HSQC.**
(BMP)

Figure S7 **DQFCOSY spectrum of zeamine and zeamine I.**
(BMP)

Figure S8 **High resolution MS-MS spectrum of zeamine I.**
(PNG)

Figure S9 **Amino acid sequence of** ***zmn5*** **encoding a phosphopantetheinyl transferase (PPTase).** Conserved motifs are underlined and indicate that Zmn5 is a PKS/NRPS-related PPTase (EntD-like) rather than PUFA-specific.
(BMP)

Figure S10 **Deduced amino acid sequence of zmn14 encoding a thioester reductase domain.** The R1 domain, involved in NAD(P)H binding as well as the five other core motifs (R2–R5) are underlined.
(BMP)

Figure S11 **Multiple alignment and active site sequences (highlighted) of polyketide and nonribosomal peptide domains.** A/Multiple sequence alignment of KS-domains. The catalytic triad is highlighted in green, conserved motifs in yellow. B/Multiple sequence alignment of AT-domains. The active site motif is highlighted in red, substrate specificity determinants in yellow. C/Multiple sequence alignment of KR-domains. The catalytic triad is marked in green, the Rossman fold involved in NADPH cofactor binding is highlighted in yellow, the residues that indicate stereospecifity in red. D/Multiple sequence alignment of C- and E-domains. The signature HHxxxDG motif is highlighted in green whereas epimerization domain-specific conserved motifs are highlighted in yellow. E/Multiple sequence alignment of PCP-domains. The conserved 4′-phosphopantetheinyl binding site motif LGGXS is highlighted in yellow. The essential serine residue in green. F/Multiple sequence alignment of ACP-domains. The conserved 4′-phosphopantetheinyl binding site motif is highlighted in yellow. The essential serine in green.
(DOCX)

Table S1 Sites of plACKO transposon insertion sites.
(BMP)

Table S2 Oligonucleotide primers used in this study. Restriction sites are underlined.
(BMP)

Table S3 Bacterial strains and plasmids used in this study.
(BMP)
