# Supplementary figures and images for: A PKS/NRPS/FAS Hybrid Gene Cluster from Serratia plymuthica RVH1 Encoding the Biosynthesis of Three Broad Spectrum, Zeamine-Related Antibiotics
Source: PLoS One. 2013 Jan 17;8(1):e54143. doi: 10.1371/journal.pone.0054143 (PMC3547906; doi:10.1371/journal.pone.0054143)

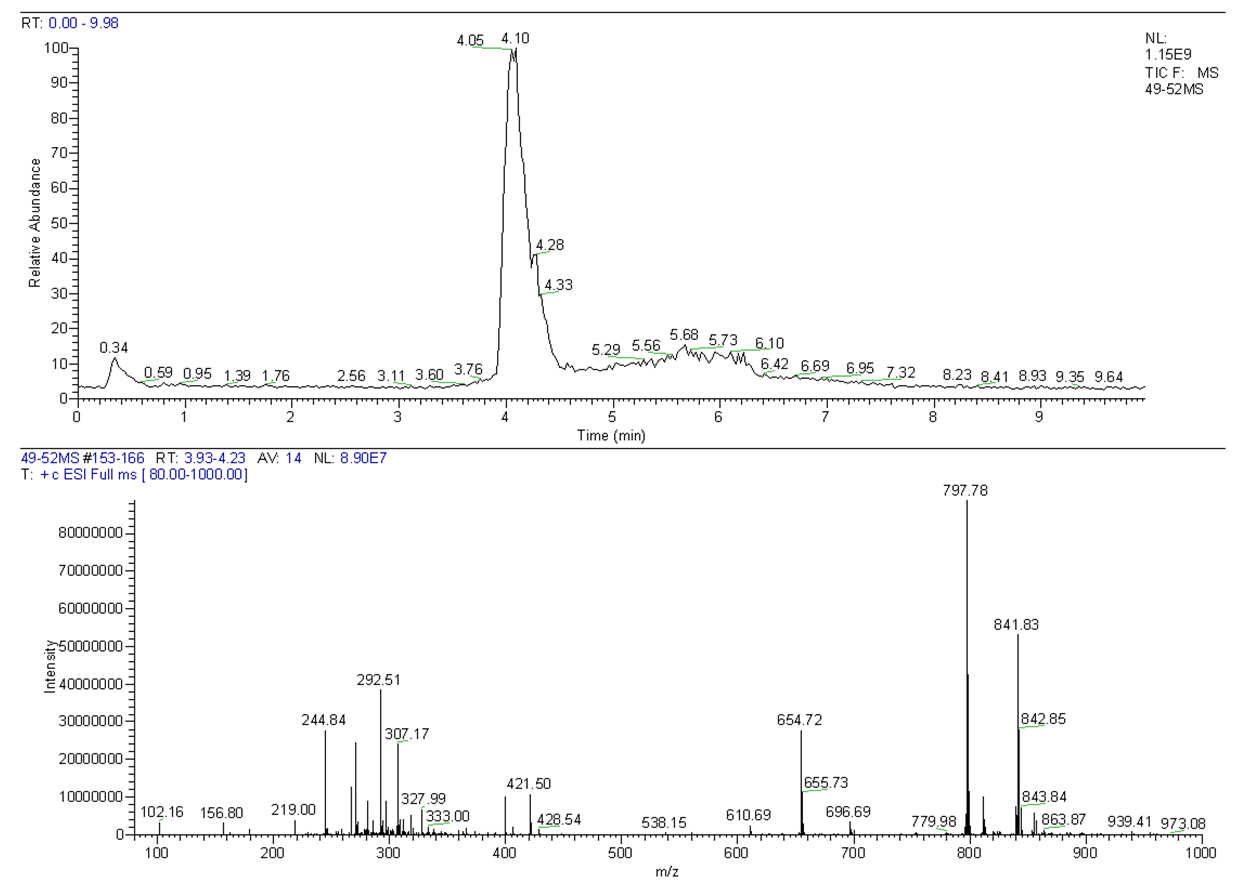

Supplement: Figure S1 — LC-MS analysis of purified extract of S. plymuthica RVH1 containing zeamine, zeamine I and zeamine II. (PNG) [file pone.0054143.s001.png]

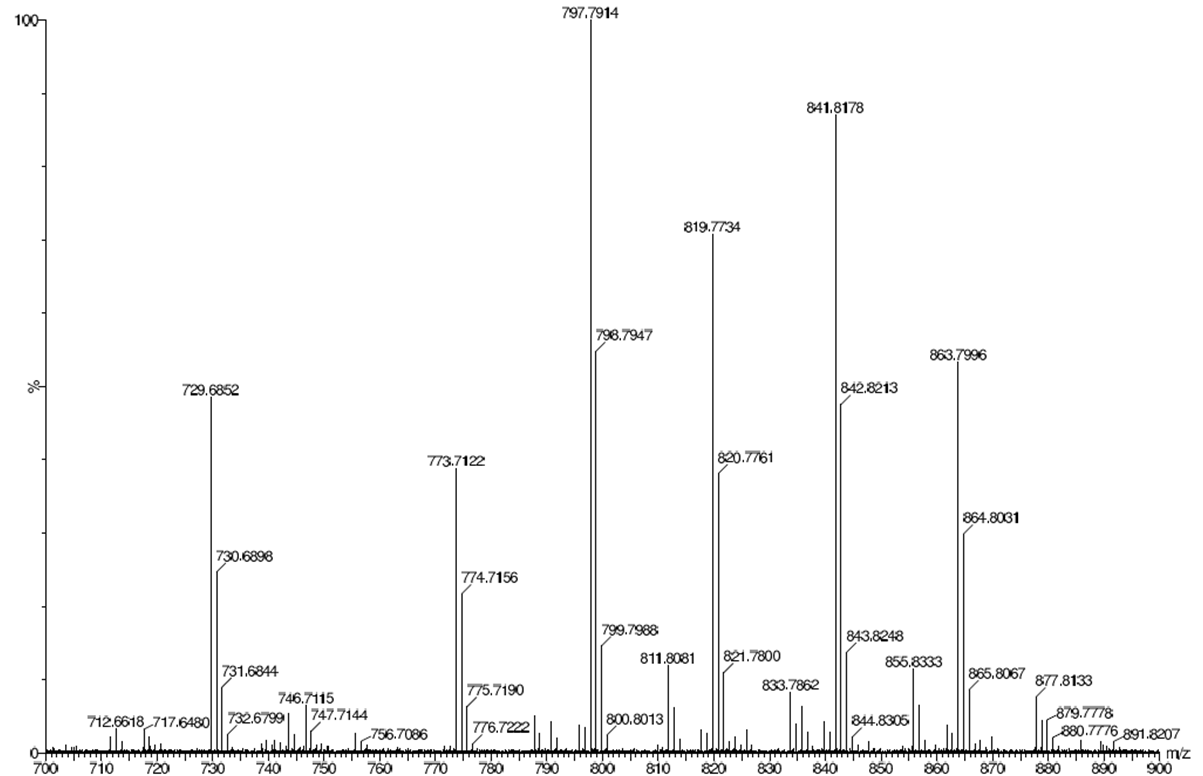

Supplement: Figure S2 — HRMS analysis of zeamine and zeamine I. (PNG) [file pone.0054143.s002.png]

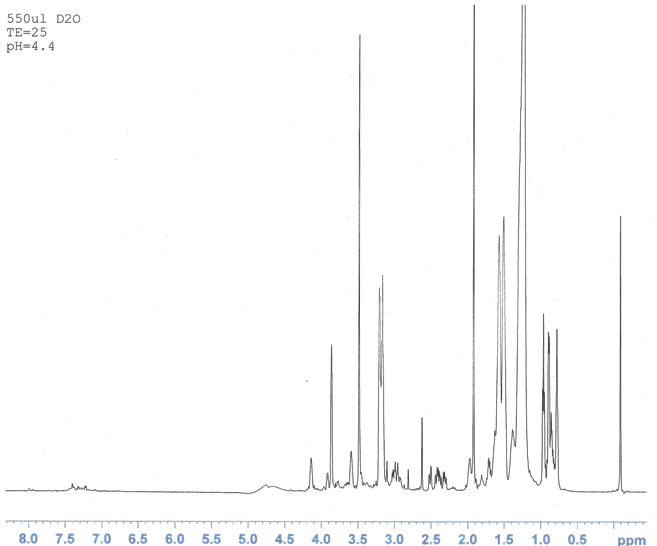

Supplement: Figure S3 — 1H-NMR spectrum of zeamine and zeamine I. (BMP) [file pone.0054143.s003.bmp]

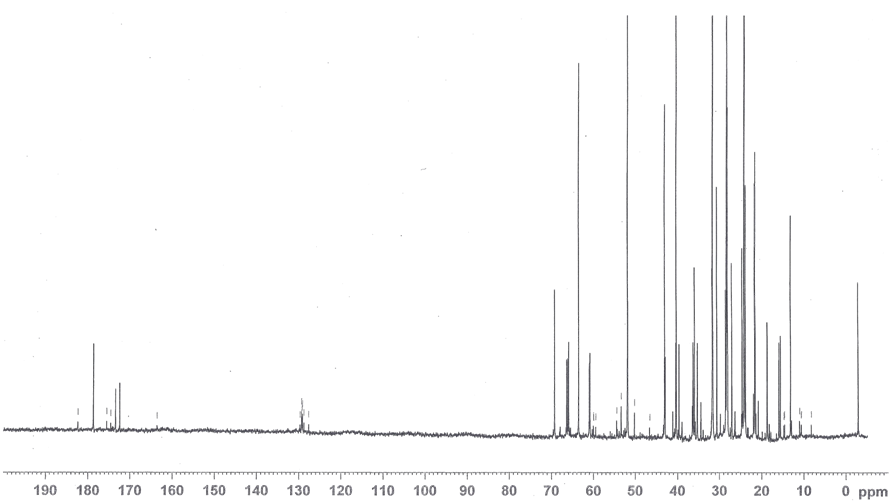

Supplement: Figure S4 — 13C-NMR spectrum of zeamine and zeamine I. (BMP) [file pone.0054143.s004.bmp]

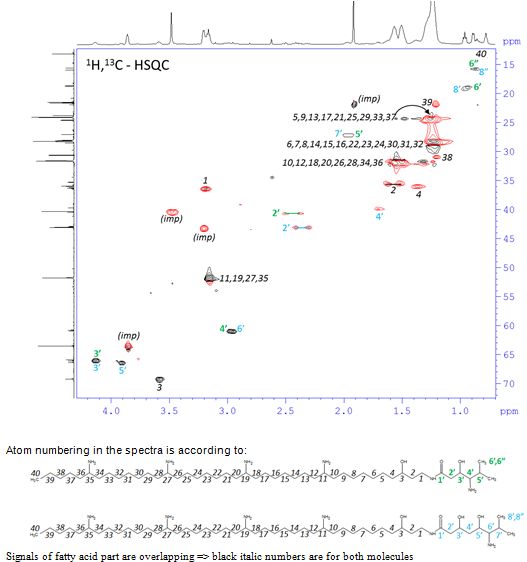

Supplement: Figure S5 — 1H,13C – HSQC spectrum of zeamine and zeamine I. (BMP) [file pone.0054143.s005.bmp]

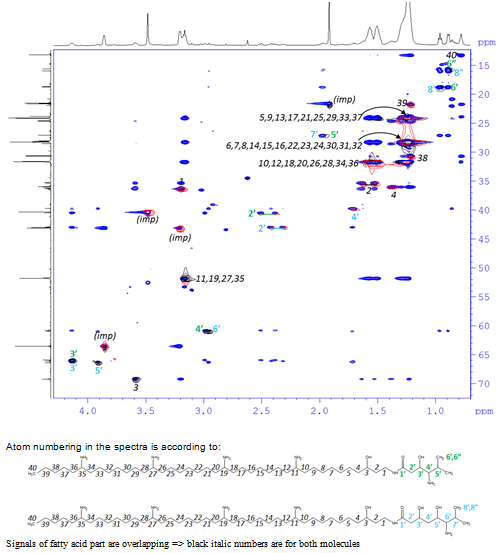

Supplement: Figure S6 — 1H,13C – HSQC-TOCSY spectrum of zeamine and zeamine I overlayed with 1H,13C – HSQC. (BMP) [file pone.0054143.s006.bmp]

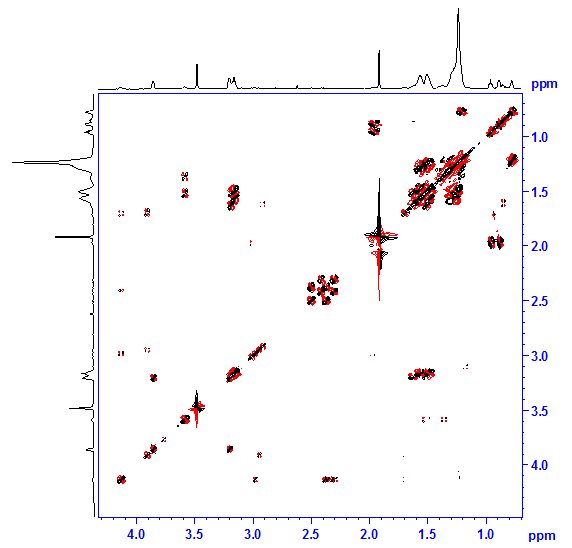

Supplement: Figure S7 — DQFCOSY spectrum of zeamine and zeamine I. (BMP) [file pone.0054143.s007.bmp]

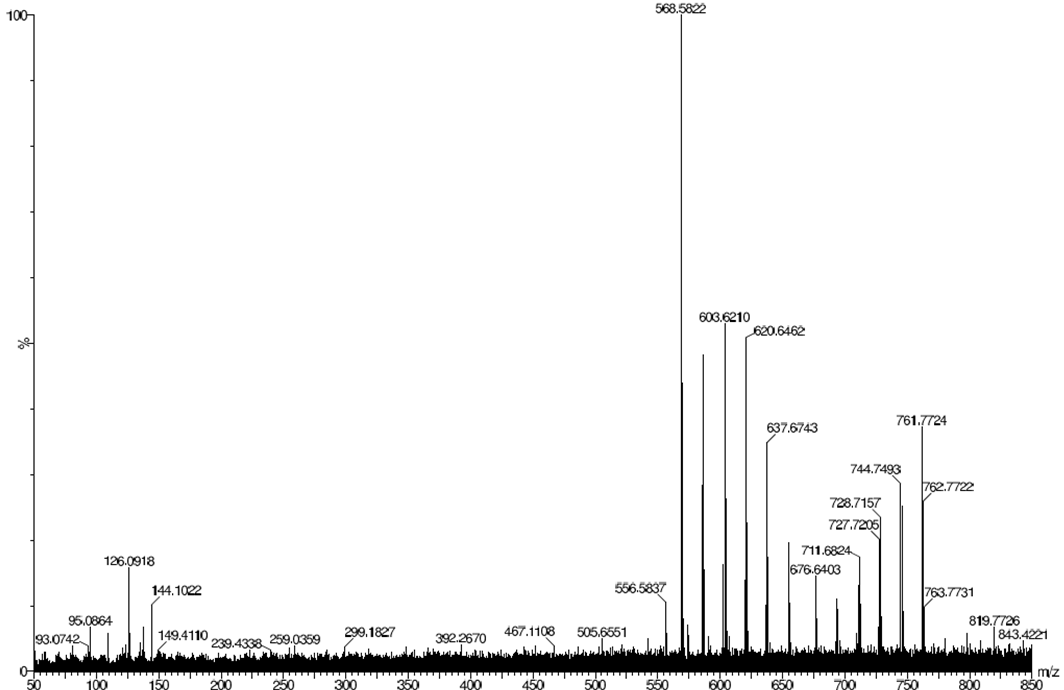

Supplement: Figure S8 — High resolution MS-MS spectrum of zeamine I. (PNG) [file pone.0054143.s008.png]

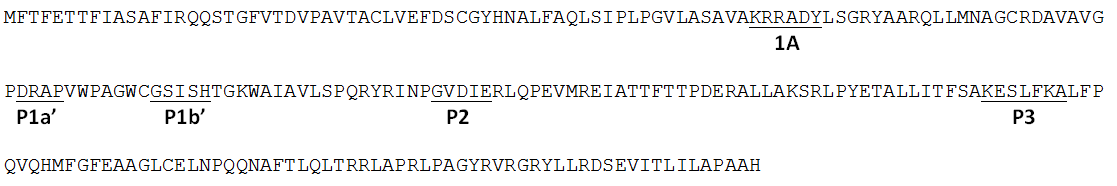

Supplement: Figure S9 — Amino acid sequence of zmn5 encoding a phosphopantetheinyl transferase (PPTase). Conserved motifs are underlined and indicate that Zmn5 is a PKS/NRPS-related PPTase (EntD-like) rather than PUFA-specific. (BMP) [file pone.0054143.s009.bmp]

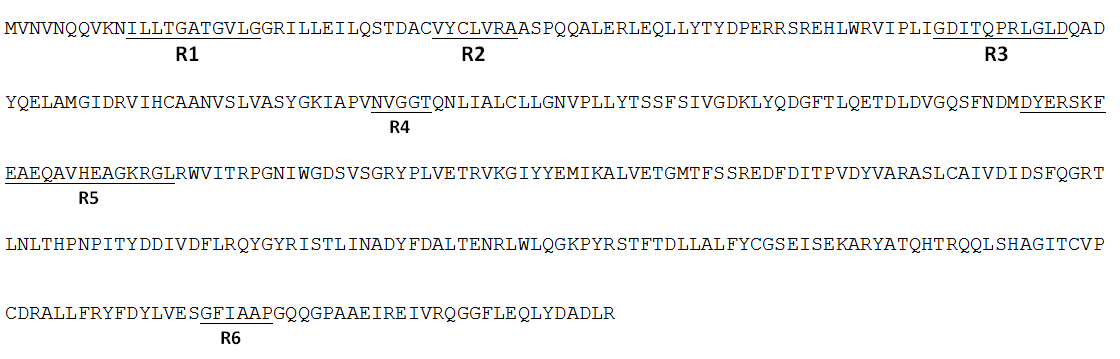

Supplement: Figure S10 — Deduced amino acid sequence of zmn14 encoding a thioester reductase domain. The R1 domain, involved in NAD(P)H binding as well as the five other core motifs (R2–R5) are underlined. (BMP) [file pone.0054143.s010.bmp]

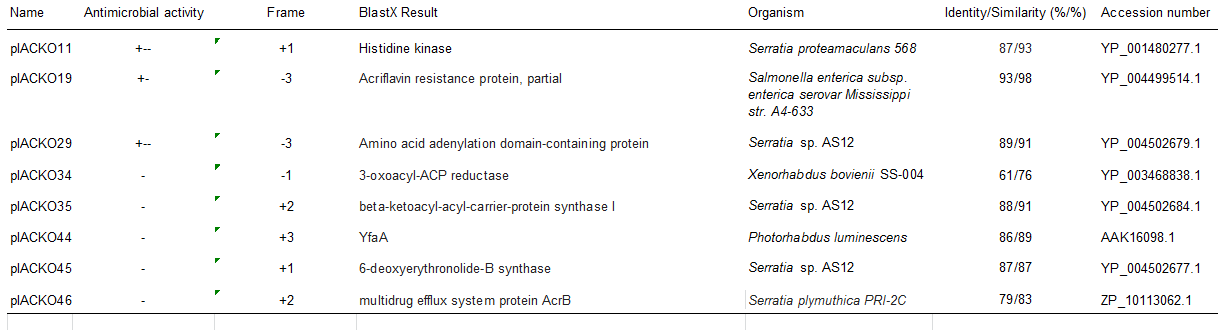

Supplement: Table S1 — Sites of plACKO transposon insertion sites. (BMP) [file pone.0054143.s012.bmp]

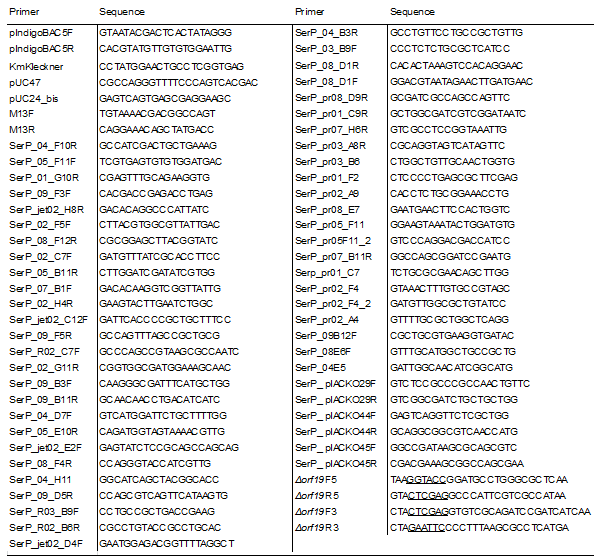

Supplement: Table S2 — Oligonucleotide primers used in this study. Restriction sites are underlined. (BMP) [file pone.0054143.s013.bmp]

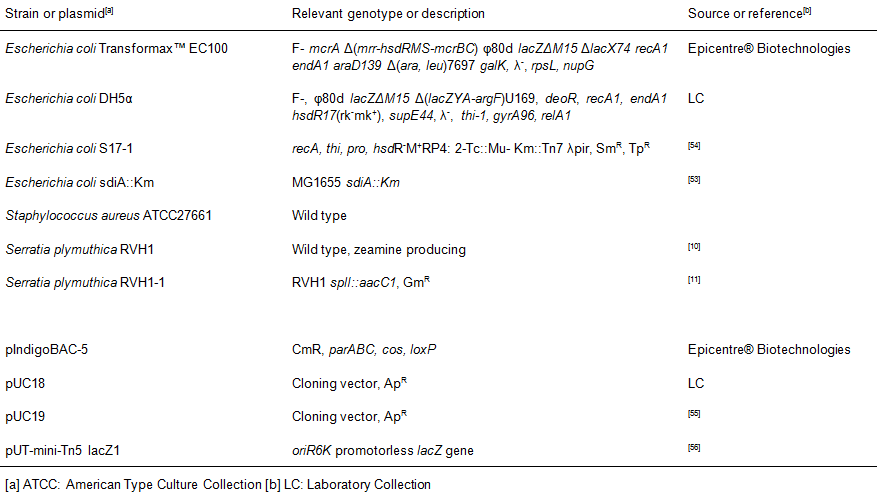

Supplement: Table S3 — Bacterial strains and plasmids used in this study. (BMP) [file pone.0054143.s014.bmp]
