# Supplementary material for: A PKS/NRPS/FAS Hybrid Gene Cluster from Serratia plymuthica RVH1 Encoding the Biosynthesis of Three Broad Spectrum, Zeamine-Related Antibiotics
Source: PLoS One. 2013 Jan 17;8(1):e54143. doi: 10.1371/journal.pone.0054143 (PMC3547906; doi:10.1371/journal.pone.0054143)
Supplement: Figure S11 — Multiple alignment and active site sequences (highlighted) of polyketide and nonribosomal peptide domains. A/Multiple sequence alignment of KS-domains. The catalytic triad is highlighted in green, conserved motifs in yellow. B/Multiple sequence alignment of AT-domains. The active site motif is highlighted in red, substrate specificity determinants in yellow. C/Multiple sequence alignment of KR-domains. The catalytic triad is marked in green, the Rossman fold involved in NADPH cofactor binding is highlighted in yellow, the residues that indicate stereospecifity in red. D/Multiple sequence alignment of C- and E-domains. The signature HHxxxDG motif is highlighted in green whereas epimerization domain-specific conserved motifs are highlighted in yellow. E/Multiple sequence alignment of PCP-domains. The conserved 4′-phosphopantetheinyl binding site motif LGGXS is highlighted in yellow. The essential serine residue in green. F/Multiple sequence alignment of ACP-domains. The conserved 4′-phosphopantetheinyl binding site motif is highlighted in yellow. The essential serine in green. (DOCX) [file pone.0054143.s011.docx]

**A**

KS1 -DIAIVGMASHFPDASNLHEFWENITNKCDSLTDISSMSDAAYWRKEDFYDPDATAADKT 59

KS4 -EIAIVGMAGSFPGAADIEAFWRNIAAGVEAIRELSDEELDAAG------TPHAEREQPD 53

KS2 ENIAIVGLGCLFPGADSTEKYWENLIQCRDCASPLSAAETGIDP--AVYYHPVAGTADKI 58

CLF EPLALIGIGVHAADTEDTLSLDRTFREGWQHQRPLPATRWLGVE-----RQQDLLATRGI 55

:*::*:. ..: . ..: : :.

KS1 YGYKAGFVP-AIEFDPVAFKIPPAIMDSISTAQLFSLYMARQVMQDAGLADKKDAQVDRD 118

KS4 FVRRAAVLEDAEAFDAAFFGYSPREAQAMDPQQRLFLETAWLALEHAGYAPGK----FPG 109

KS2 GYNRNGHIR-DFHFDTSGYALPAQELETLDNLFKWTLHAAAQALNDSGYREQPQTLAKTG 117

CLF EQIPQGAWIETFEFDCKRFRLPPNVVGPHLLSHLFLLPVAERAFIDAGYALDG------- 108

. ** : .. . * * .: .:*

KS1 RIG--VILGGGGNGNTSFSLAARQQAPYLREIMIKSGLSAE--------VADDVIARAHE 168

KS4 QIG--VFASAS---NSSYAMSYLLRHPDIQRHVALDGLLSG--------NNNDLLS---- 152

KS2 LLIGNIGMPTHSGKRLMSRFYHQMLTPYLRQLLGRDDFAFDRVWPEETLSDLN------- 170

CLF -----------TRRNLAVIVAGGVDYSCLRYQAR-------------------------- 131

. . . ::

KS1 LYLEWNEDSFPGFLGNVACGRIASYFDLGGTSYMVDAACASSLAAIKAAIGELNSGSCDA 228

KS4 -------------------TRVAYKLNLRGPAMTIGAACSSSLLALHAACQSLLAGECDM 193

KS2 -----------LITGGHNATVAAQALGLQGPSYALDAACSSAFYAIKMASDYLASGKADM 219

CLF -------------------NEISWQLEQSLRSQGIALSEEDLQALQNVVKDSLFPAPYPE 172

: : : : : . : . * ..

KS1 VLTGGVNLENSAFSFLCFSKTPALSKSNRSRPFDRDSDGIMLGDGVGLLVLKRLEDA--- 285

KS4 ALGGGVHVGFPLKSGYRYENSGILSHDGRCRSFDADASGTVAGDGVGAVVLKRLQDA--- 250

KS2 MLAGAVCHADHIYIDHGFNVLQAFPQQGQSIPFDRTSEGLKAGEGAGVIAMKRYADA--- 276

CLF GITGGIGNVVASR------LAAHLHLNGPAFSLYSEENAAFKAIELAQFLLARHEVEGVL 226

: *.: : .. . .: .. . . . : *

KS1 YAVIKSLEASSDGRAKS-IFAPRYEGQIKALKRAYASAGILPGDIQLIEAHGTGTASGDG 344

KS4 YAIIKGSAVNNDGSSKPGFTAPSINGQTEVLRRALVAADVAPESIGYIEAHGTATRLGDP 310

KS2 YGVIEAVGLSNDAGSRH-MLVPDSNGQQLALTRAYG---KAEPDIDYLECHATGTPVGDQ 332

CLF YAVIDGLAVGHELSPALAYTASR-QGIADSASRALQAAGCSAADVDYLELYSGARPGEME 285

*.:*.. . : . .. :* ** .: :* :. .

KS1 TELKSLRAVFDEYRLPAHSIAVGSVKSQIGHTRCAAGAASMIKVALALHHKVLPPSIN- 402

KS4 IELKALLNAYGKQRGPDHRCAVASVKANVGHLDSAAGVTGVIKVAHMLMHRYLPPAVNF 369

KS2 IELAAMEAFFAQAP---TRPLLGANKGINGHMLTASGMGSLFKVILAMQHNIIPPTPG- 387

CLF TELAALAGVYATPE-RQVPVTVGTLKAHYGHLAAAAGLLGIVKCALQLSQRYLPAV--- 340

** :: : :.: *. ** *:* .:.* : :. :*.

**B**

AT1 --FPGQGSQYVNMGREVAIDYPEMRQALETLDKFAADVRGHGLSDIVYPTPAFSDAERDA 58

AT2 FMFSGQGSQYAGMMHDHYQQEPFFRDVVDECAGLLKPLLGRDIR------PLLFEAADDA 54

*.******..* :: : * :*:.:: : : *:.: * : :* **

AT1 QRERLTDTANAQPAIGAVSAGYYAILKSNGFTPDFVAGHSYGEVTALWAAGALCDADFYR 118

AT2 SLSALYQTRFTQPALFVVEYALSRLLMSWGLAPAACIGHSIGEYVAACLAGVFSLEDGLR 114

. . * :* :***: .*. . :* * *::* *** ** .* **.:. * *

AT1 ASLARGMAAASAVAQNEGDGGAMLAAALTVEACEKLLVRYPDIVLANDNSPAQVVLGGAT 178

AT2 IVVRR------AALMNEMPPGKMLAVGLSAAELARWLP--DGVSLASENAPEFCVASGES 166

: * *. ** * ***..*:. : * .: **.:*:* * .* :

AT1 ARIHALYDELKAGDVQCRILPVSAAFHTPFLQAACAPFLESLSSIRFDTTHCTAFSSASA 238

AT2 EAIETLQALLDARGVEAKPLHTSHAFHSAMMEGCLADFERAFDDITLSPPGLPFISCVTG 226

*.:* *.* .*:.: * .* ***:.:::.. * * .::..* :... . :*..:.

AT1 APWPADTATYAETLAQQMIMPVRFRETIEALYQRGGRLFVEIGPKGVLGKLVADILKGRE 298

AT2 DWIRPEQATSPDYWVSQLRQPVAFSQGVKRLLDEPQNLLLEVGPGSTLTGL--------- 277

.: ** .: ..*: ** * : :: * :. .*::*:** ..* *

AT1 HTLISLNTGDSGDDRLQLARAQARL 323

AT2 -------------------------

**C**

KR1 --LVVTGGARGITAKCVIELAQRSQAHFILLGRTDIDAPLPAWAQGTATVAERKAAAIAQ 58

KR3 GNYLITGAFGGIGSTIAENLARRHRARLLFIAR----GELPPEAEWESWLAQHDASNAKS 56

KR2 -VAVVTGAAGGFGTAIARVLLDIG--------------------------YQVAAADVSA 33

::**. *: : . * : *:

KR1 LQSEGVQPTPVKIDAMLSGLLHSDEINATLHQIAQAGGEAIYRHCDITDAQQVATVLNEA 118

KR3 RR------------------------IAFLRQLQQAGAQVTVISADLADKRSVGRGIAAF 92

KR2 ER------------------------LTQLAERLGHPEGLHTFVMDVTQEESIAQAAREI 69

: : * : *::: ..:.

KR1 QQVVG-PITGIIHGAGNLADKRIE-KKTLGDLRSVFDVKVRG----LENLCRALDITGLR 172

KR3 TQRYG-TLNGIFHCAGVADGGLIQ-NRSADDSQRVFQAKVQG----TRVLEACLRQHAPD 146

KR2 EARLGAALTVLVNNAGVIERSFCLSERGLSGAARVLNVNLLGTFNCTAVFSRYMARLKYG 129

* .:. :.: ** :: .. *::.:: * : :

KR1 HVMLFSSVSGFFGNAGQTDYAMANETLNKFAWLPLQT-EGQPTVRAINWGPWDG- 225

KR3 FFVVCSSLAAFVGPVGQVAYCAANAWQDAWALNAHQRRDARTRYLAIGWDAWRDV 201

KR2 RIINIASIAGIWGAAGGSAYAAS---------------KAGVISATESWGREL-- 167

.: :*::.: * .* *. : .. : .*.

**D**

C1 QSSVTLSDEQQGMWLIQQQAPESPVWQLAYRAEWQG--PLNIELLQQALNQALRRHDALR 58

C4 ----RASLAQKRLWLHEQLGHDSAIYHISTLLELEG--ELDETALQQSVNDLVARHESFR 54

C2 --EAPLSFAQQRIWFLEQLHPDGNSYITGEIKTFTAP-AVTIERLSAALDALFRHFPLLN 57

C3 -DIYPLTPTQEGMLYHSLADGDARLYHEQICFDLAR--GVDLARLQQAWAQVIAACPALR 57

C5 -DAYPLSRMQAGMFYHMNLTPDANVYHCTGTSHLRINAAFDEAAFRRAVAETVAAHDVLR 59

C6 --VYPLGYAQHCFWFVAATLGDTANNQS--CIQIEG--ELQPALLERVLGGLIAHHPILR 54

E -QAYKLLPAQR--WFFALGAEKAHHYNQSYLWRLDA--QVNLPQLERALRGVLQQHEGLR 55

* . . : . :.

C1 TRFIPDQDG-VTAQIAEQVVVDIDRQDFGAQGSDA-RGAAEGWLTQLATRPFNLAQAPLL 116

C4 TDFVWQDEA-LFQRCHDTMTLPLEKVDWVAAD----EAALQAQLNAASERPFNLQQAPLS 109

C2 TRLRADG----AVPCQYWSEAPVEFTAEGVDDDAW-HACVQQRAAELMSEPFTLLAAPLY 112

C3 TTFDLNAEAGPLQLVHQQMPDAWRVVDYRSPQ----EEDIAALWRADREQGFDLHQGPLF 113

C5 TGFDFARYSQPLQLVWPQAELPIVVEDLCHLDKQRQEERIRALLDEERRTPFDLQRPTLL 119

C6 SAISRWAPT---QRIQPVGGFDLPFADLTGETPVQAQQHVKDVARRLLAEPFDLQRPPLL 111

E ARFSAADAP---ATIAALTDAPLLRETCLGAP--HDDEAAVIAWAGARQSQLDIAQGPVF 110

: : : : .:

C1 RIACAQVQPHRTLIALVCHHIIADAESVALLWRQMLADYGRLLAGKTLTPVAATACQTAH 176

C4 RFVLFRRGACHHMLLIVLHHIISDGWSAGIISRELASCYNARRQGLAADLPPLDIQYIDY 169

C2 RLCFIHSPQGQSALIFCVHHLLVDEHTLSLIDRSLTLLIDDPQ----AACTAPAVSYLQY 168

C3 RITVLLAQDS-VQMLFSHHHAILDGWSVTLLLAQVAAAYHQTP------PLPSAPAFRHY 166

C5 RFFIQLRSPQSFQFTMTECHPVFDGWSYHTMIVEVFNRYAVLTGG--SQWQPPVKPQQEY 177

C6 RAQLFRLQPQQHVLVLCFPHIVADGAAVHLFCQQLWQRYRLLARGDKLPLAGQAEMPFTA 171

E SADVIRAASADYLLLS-VHHLVVDGVSWRIIISDLSRAYQALARGEALPCEPATASLAEW 169

: * : * : : .:

C1 ARRQQQRSAGPRGEALRAWWRQQLAGPLPLLQLPYDFPVPAVRSYCGGRIPFHLSQATAG 236

C4 SEWQHQQQEGDDVARSLDWWREKLRN-LEPIDLPCDRPRPNRLSLRGKTRFFSLPPALAQ 228

C2 ATWERSTSHQEG----LDWWREVFLTPPPVIALPQ-RPQPDETDWRAAYQTLTLPLPLTE 223

C3 IQWLRRSRAGSQ-----AHWQQRLAGLSAATPLPAAYPGRTPSRQR-QRQDFQLDAALSA 220

C5 RDFIVQEQAAIRDDAQRRYWQQTLADCTLLRLPHQAQDATPGTAPRLKSLSFTLDDTVYQ 237

C6 LIASERARYRRHGSEDQRFWRRHLQGYPWATFPQCYVNQQQ---THASDRYVSFPADSYE 228

E GAWLQQVVDPVSAADEHRYWETQRHAGAAIPVDSGMRALPGRTGEMRKVQITLDRAATQA 229

*.

C1 ALAALAVRHGVTPYVALLTAYTAFMARYTRQQDLTIGTPISSRHALD----NEQAFGLLF 292

C4 TLGERARARNSTLYSLLLSAFSLILHRYSGQRDLAIGSPVSGRGRLE----LEGVVGFFV 284

C2 ALRQLARQQDTTLFTLLLSTFQLLLQRYSGENDLTIGLPVSLRDSDT----LQNLPGLLL 279

C3 QLARFGRRHRLTISTLVQGAWALLLQRYSGSTESLFGVTLSGRHADLSG--IERMVGLLI 278

C5 GMRQLMQQLGVPLKSVLLAAHVKVMSVFSGEPDILTGIPTNGRPEAE-G--GDWLYGLYL 294

C6 RLESVARQHKVSLQMVLLALIGRVVREMTGASRFALNSVLEGRDRPG----SESLMAPLL 284

E LVRQTGRAYHTHVGDLLLAALAQTLSEWTGSRRLRIDFEGHGRDDAPDAPDVSRTVGWFT 289

: : : : . * . .

C1 NVVALRQTV---- 301

C4 NTLVYRIQID--- 294

C2 NTLAFRQTL---- 288

C3 GTVPMRASVQPDV 291

C5 NILPFR------- 300

C6 RVMPVPLDM---- 293

E AIYPILLQLPSTG 349

**E**

PCP4 -AIQATWGEILRCGPVSCDQNFFALGGHSLHALRVAARLENQLNVRVSLTDLLNHQTVRQ 59

PCP6 -AIQQVWSKVLGVAAPNQQENFFDLGGDSLLLARAHLRLAALGYSGVSIVDLLNYPTIQQ 59

PCP3 -QLAAIWQKVLGGETPGMDDDFFALGGDSILSLQIVFLAREAG-LRVQARMLFDYPTIAS 58

PCP5 -ALLALWRDVLKVDDIGVYDNFFSLGGDSLRGVQVVGRGRERG-LALTLVNLFQHQTVAE 58

PCP2 QAIAAVWSRVLGIEGIGRDDSFFALGGHSLLAARAVALLAAEHQLHLRVSDIFTRRTPRQ 60

PCP1 QKLAAIWQRLLPVNAVGRDDDFFALGGNSLLAMRMIACVRAELDRELEFAALLAQPTLAG 60

: * :* . :.** ***.*: : : :: *

PCP4 LAAV- 63

PCP6 LADYI 64

PCP3 LAS-- 61

PCP5 LAALL 63

PCP2 LA--- 62

PCP1 -----

**F**

ACP1 RLVTIVSDRTGYP-QDMIDANMDLEADLGIDSIKRLEIFGAMFDAFS----------------- 46

ACP2 ----QITAAWQEKMGIGPLGIEDDFFELNGHSLMAVQIIALIRQRLNVSLPVGFIYDHPTVAAL 60

ACP1 --- 46

ACP2 SEQ 63

Figure S11: Multiple alignment and active site sequences (highlighted) of polyketide and nonribosomal peptide domains

**A** Multiple sequence alignment of KS-domains. The catalytic triad is highlighted in green, conserved motifs in yellow.

**B** Multiple sequence alignment of AT-domains. The active site motif is highlighted in red, substrate specificity derterminants in yellow.

**C** Multiple sequence alignment of KR-domains. The catalytic triad is marked in green, the Rossman fold involved in NADPH cofactor binding is highlighted in yellow, the residues that indicate stereospecifity in red.

**D** Multiple sequence alignment of C- and E-domains. The signature HHxxxDG motif is highlighted in green whereas epimerization domain-specific conserved motifs are highlighted in yellow.

**E** Multiple sequence alignment of PCP-domains. The conserved 4’-phosphopantetheinyl binding site motif LGGXS is highlighted in yellow. The essential serine residue in green.

**F** Multiple sequence alignment of ACP-domains. The conserved 4’-phosphopantetheinyl binding site motif is highlighted in yellow. The essential serine in green.
